# Exploring the Potential of Bimetallic PtPd/C Cathode Catalysts to Enhance the Performance of PEM Fuel Cells

**DOI:** 10.3390/nano14201672

**Published:** 2024-10-18

**Authors:** Vladimir Guterman, Anastasia Alekseenko, Sergey Belenov, Vladislav Menshikov, Elizaveta Moguchikh, Irina Novomlinskaya, Kirill Paperzh, Ilya Pankov

**Affiliations:** 1 Faculty of Chemistry, Southern Federal University, 7 Zorge St., Rostov-on-Don 344090, Russia; aalekseenko@sfedu.ru (A.A.); sbelenov@sfedu.ru (S.B.); vmenshikov@sfedu.ru (V.M.); moguchih@sfedu.ru (E.M.); igerasimova@sfedu.ru (I.N.); paperzh@sfedu.ru (K.P.); 2 Prometheus R&D LLC, 4g/36 Zhmaylova St., Rostov-on-Don 344091, Russia; 3 Research Institute of Physical Organic Chemistry, Southern Federal University, 194/2 Stachki St., Rostov-on-Don 344090, Russia; ipankov@sfedu.ru

**Keywords:** palladium, platinum alloy, nanoparticles, catalyst, electrocatalysis, PEMFC, oxygen reduction reaction, membrane electrode assembly, mass activity, durability

## Abstract

Bimetallic platinum-containing catalysts are deemed promising for electrolyzers and proton-exchange membrane fuel cells (PEMFCs). A significant number of laboratory studies and commercial offers are related to PtNi/C and PtCo/C electrocatalysts. The behavior of PtPd/C catalysts has been studied much less, although palladium itself is the metal closest to platinum in its properties. Using a series of characterization methods, this paper presents a comparative study of structural characteristics of the commercial PtPd/C catalysts containing 38% wt. of precious metals and the well-known HiSpec4000 Pt/C catalyst. The electrochemical behavior of the catalysts was studied both in a three-electrode electrochemical cell and in the membrane electrode assemblies (MEAs) of hydrogen–air PEMFCs. Both PtPd/C samples demonstrated higher values of the electrochemically active surface area, as well as greater specific and mass activity in the oxygen reduction reaction in comparison with conventional Pt/C, while not being inferior to the latter in durability. The MEA based on the best of the PtPd/C catalysts also exhibited higher performance in single tests and long-term durability testing. The results of this study conducted indicate the prospects of using bimetallic PtPd/C materials for cathode catalysts in PEMFCs.

## 1. Introduction

Hydrogen–air fuel cells with a proton-exchange membrane (PEMFCs) are used as the main source of energy in various types of devices, including but not limited to electric cars, scooters, passenger cars and trucks, undersurface and surface vessels, trams and buses, drones, copters, and even civil aviation aircraft [1,2,3,4,5]. In addition, PEMFCs are successfully used for power supply to stationary consumers, including cell phone towers, stationary generators for social facilities and units where continuous power supply is critical, private housing, etc. [1,2,3]. Pure hydrogen, which is used in PEMFCs as fuel, is usually obtained using electrolyzers. Electrolyzers with a proton-exchange membrane (PEMEs) are playing an ever-growing role in this production [6,7,8]. To reduce overvoltage and accelerate cell reactions at the PEME cathode and at both PEMFC electrodes, platinum-containing electrocatalysts are used [7,9,10,11].

In the mass production of PEMFCs, the cost of catalysts value is going to amount to 30–40% of the cost of membrane electrode assemblies (MEAs) [12,13], and in the production of PEMEs that use iridium catalysts at the anode, this percentage is seen to be even more. Therefore, the task of reducing the amount of platinum in catalytic layers is still deemed quite relevant.

Platinum-containing catalysts represent nanostructured composites containing nanoparticles (NPs) of platinum or its alloy deposited on micro-/nanoparticles of a dispersed electron-conducting support, which is most often carbon [14,15,16,17]. It is noteworthy that other metals themselves cannot compete with platinum in terms of catalytic activity in electrochemical reactions proceeding in PEMFCs [16,18]. The catalyst’s high activity in the oxygen electroreduction reaction (ORR) is particularly important, since it is this reaction that makes the main contribution to the overvoltage of PEMFCs.

Controlling the shape and size of NPs, as well as the uniformity of their spatial distribution, including alloying platinum with other metals, can significantly increase the catalyst activity [16,19,20,21,22] (see below). A mixture of the platinum–carbon catalyst, a proton-conducting polymer of the Nafion type, and a solvent is applied to the surface of a proton-exchange membrane or an MEA gas diffusion layer to form a porous catalytic layer after drying [15,23,24,25]. Reducing platinum loading in the catalytic layer with no decrease in its characteristics is possible by optimizing the design of the layer itself [24,25]. The purpose of such optimization is to increase the electron and proton (due to the macromolecules of the proton-conducting polymer) conductivity of the layer, reduce the proportion of platinum NPs not included in the electric current generation process, and facilitate mass transfer of reagents and a product (water) in the layer.

Another way to reduce the loading of platinum in the catalytic layer is to enhance the specific characteristics of the catalyst itself, which may be conducive to an increase in its ability to convert a larger amount of reagent per unit time. Essential characteristics of the catalyst are the specific electrochemically active surface area (ECSA) and the specific activity of the metal component *(I_sp_)*, as well as the catalyst stability that reflects its ability to operate for a long time with no decrease in its ECSA and *I_sp_*. An increase in the ECSA of the catalyst is possible by reducing the average size of NPs, although this may result in a decrease in *I_sp_* and catalyst stability [26,27,28]. Optimization of the NPs’ shape can also lead to an increase in activity, since different parts (facets, edges, vertices) of platinum nanocrystals may exhibit different specific activity [29]. The activity of the catalyst is often measured per unit mass of platinum. Mass activity *(I_mass_)* makes it possible to actually account for the influence of the ECSA on the *I_sp_* value. In a simplified form, the relationship between mass activity, specific activity, and ECSA can be expressed by Formula (1) as follows:

*I_mass_* = *ECSA* × *I_sp_* (1)

The simplification of Formula (1) is due to the fact that the *I_sp_* value is an averaged parameter of some kind, the value of which is precisely determined by the contribution of different (in activity) sections of the metal surface to the total ECSA [30]. Due to the two differently directed trends, the curve of dependence of the ORR mass activity on the average size of NPs has a maximum. The position of this maximum determines the size range of platinum-containing NPs, which are characterized by the highest mass activity (the so-called “optimal size”). The identification of the optimal particle size is still an urgent task, since the relevant data obtained by various researchers are rather contradictory [31,32,33,34,35].

Another, perhaps more effective, way to increase the specific and mass activity of platinum is its alloying with other metals [16,36]. Although cobalt and nickel are most often used as the alloying component, many other d-metals can also act as the promoters [16,21,36,37,38]. As a rule, we are talking about the formation of NPs with a more or less uniform solid solution of the alloying component in the platinum crystal lattice [15,16,21,22,27]. The positive effect of the alloying component on the catalytic activity of platinum is associated with the following [15,16,27,32,33,34,35,36,38,39]: a reduction in the nanocrystal interatomic distance, which facilitates the adsorption of oxygen molecules on the surface of the catalyst during the ORR; a change in the energy of free d-orbitals, which also facilitates the O_2_ adsorption on the surface of NPs; an increase in the number of grain-boundary defects, and an increase in the ECSA, including the defectiveness of the NP surface due to the selective dissolution of the alloying component during the operation of the catalyst.

Bimetallic PtM NPs may have a complex structure due to a non-uniform distribution of the atoms of the alloying component in the body of the NPs, i.e., Pt-shell—M-core, onion-like structure, skeleton-type structure, etc. [32,33,34,35,38]. It is important that in those complexly arranged NPs, the atoms of the alloying component can significantly increase the specific activity of platinum. As a result, even with a relatively small ECSA value, the mass activity of the bimetallic catalyst determined by Formula (1) can be significantly higher than that of the Pt/C analog with the same precious metal loading [32,33,34,35].

Unfortunately, the significantly lower thermodynamic stability of most alloying components, compared to platinum, leads to their gradual leaching from bimetallic NPs [16,34]. This applies both to NPs with a solid-solution structure and to core–shell structures. As a result, both of them, after some time, either turn into single-component platinum NPs or transform into secondary core–shell particles coated with a relatively thick shell, mainly consisting of platinum atoms and containing a certain number of atoms of the alloying component in the core [16,40]. The stability of the composition, structure, and shape of bimetallic NPs is related to the nature of the alloying component, the initial structure of the NPs, and the operating conditions of the catalyst, including the conditions of its pretreatment [41,42].

There is one more essential aspect that should be considered when developing novel compositions and structures of two- and multicomponent platinum-containing electrocatalysts, i.e., oxygen reduction reactions. The indicators of high activity of the materials that researchers obtain when studying a thin catalytic layer in laboratory tests at a rotating disk electrode (RDE) are not confirmed during testing in the MEAs. When switching to testing in the MEAs, the same materials exhibit much lower activity values comparable to commercial Pt/C analogs [43]. Therefore, there are factors that limit the implementation of very high activity in the ORR when using the electrocatalysts in real MEAs, and, thus, the pursuit of extremely high activity characteristics upon testing the catalysts at the RDE partially does not make much sense.

Palladium occupies a special place among the components used for alloying platinum. It ranks second after platinum in terms of specific activity in the oxygen electroreduction and hydrogen electrooxidation reactions [44]. The palladium crystal lattice parameter is closest to that of platinum, which indirectly indicates a simplification of the alloying process in the Pt–Pd system. Palladium is inferior to platinum in terms of resistance to degradation [45,46,47]. However, being a precious metal, palladium has a higher thermodynamic stability compared to Ni, Co, Cu, Fe, etc. At the same time, the price of palladium is comparable to that of platinum, which reduces the attractiveness of its use. On the other hand, the partial replacement of platinum with palladium in electrocatalysts for PEMEs and PEMFCs, provided that the palladium-containing catalysts demonstrate high performance, may lead to an expansion of natural reserves of precious metals that can be used for the production of electrocatalysts.

Another interesting property of palladium in terms of its possible use in bimetallic catalysts is its density, which is almost two times lower compared to platinum [48]. This means that to form the same quantity of NPs of the same size, half the weight of palladium should be used compared to platinum. In this regard, the replacement of platinum catalysts with two-component platinum–palladium systems may lead to a decrease in the loading of precious metals in the catalyst and the MEA catalytic layer with no reduction in the total surface area of the NPs.

There is a series of papers concerned with the electrochemical behavior of PtPd/C catalysts in the hydrogen oxidation reaction (HOR) and the ORR [37,45,46,47,49,50,51]. Based on the results of these studies, it can be concluded that the catalysts based on bimetallic PtPd NPs may be more active in the ORR than Pt/C (see Table S1 in Supplementary Materials). Their activity depends on both the composition and the size and shape of the NPs. The structure of PtPd NPs in those catalysts can be different [37,49,50,51,52,53,54], i.e., alloy, nanoclusters, dendritic PtPd, and core–shell structures. Unfortunately, a direct analysis of the effect of the structure of platinum–palladium NPs on the ORR activity is impossible due to differences in other essential characteristics of the NPs and the catalysts containing them. It is worth noting that researchers report insufficient stability of PtPd/C catalysts at high potentials or under stress-testing conditions due to selective dissolution of palladium [45,46,47]. In this regard, the Pd-core—Pt-shell structure appears to be most promising, in which the platinum shell performs a protective function in relation to the palladium core [53,54,55,56].

In general, the number of papers related to the preparation and study of the behavior of PtPd/C electrocatalysts represents only a small proportion of publications in the field of Pt-alloy catalysts. Questions on the optimal composition and structure of platinum–palladium NPs and electrocatalysts based on them, as well as the feasibility of their practical use in PEMFCs and PEMEs, are still to be addressed. On the other hand, PtPd/C catalysts are presented as commercial products on the websites of some manufacturing companies [57,58]. The PtPd/C catalysts are also believed to be of interest for practical use as an alternative or supplement to Pt/C catalysts, provided that their stability and/or activity in cell reactions turn out to be higher than those of monometallic Pt/C analogs.

The purpose of this work is to compare the structural and morphological characteristics and the electrochemical behavior, in particular, ORR activity and resistance to degradation, of commercial platinum–palladium electrocatalysts manufactured by PROMETHEUS R&D (Rostov-on-Don, Russia) [59] with a well-known and commonly used Pt/C analog. At the same time, the comparative study of the catalysts’ electrochemical behavior was carried out not only in a three-electrode electrochemical cell, as used by most authors, but also in the MEAs of hydrogen–air fuel cells.

## 2. Materials and Methods

### 2.1. Materials

The PP1 and PP2 PtPd/C electrocatalysts are experimental commercial products manufactured by PROMETHEUS R&D LLC (Russia) [59]. According to the manufacturer, both catalysts were obtained by liquid-phase synthesis. In this regard, the PP1 sample was synthesized by reducing precursors to metallic NPs in the reaction medium containing a mixture of Pt and Pd precursors. The PP2 sample was produced through the sequential reduction of palladium and then platinum from the solutions of their precursors. The ratio of metals in NPs, calculated by the manufacturer based on the assumption of complete reduction of their compounds to the metal during synthesis, corresponds to the formula Pt_3_Pd_1_. Vulcan XC-72 was used as the carbon support.

The mass fraction of metals in PP1 and PP2 was 37.5 ± 1.1% wt. and 38.2 ± 1.1% wt., respectively. Therefore, a widespread commercial Pt/C catalyst with a 40% loading of platinum (HiSpec4000 (Johnson Matthey, London, UK)) was selected as the conventional sample.

For information about the reagents used, see Supplementary Materials, Section S1.

### 2.2. Study of the Catalyst’s Composition and Structure

The mass fraction of metals in the catalyst was determined by thermogravimetry, with an accuracy of 3% wt. The composition of the metal component was determined by total reflection X-ray fluorescence (TXRF) and inductively coupled plasma atomic emission spectroscopy (ICP AS). The phase composition, as well as the average size of PtPd crystallites were determined by the X-ray powder diffraction (XRD) method. The size dispersion, average size, and structure of NPs were assessed by transmission electron microscopy (TEM), high-angle annular dark-field scanning transmission electron microscopy (HAADF-STEM), and scanning electron microscopy (SEM). The accuracy of determining the size of crystallites and NPs was ±10%. The composition of the catalyst’s local sections was studied using energy-dispersive X-ray spectroscopy (EDX). For details on using the structural research methods, see Supplementary Materials, Section S2.5.

### 2.3. Electrochemical Methods of This Study

The electrochemical behavior of the catalysts was studied by cycling and linear voltammetry using the VersaSTAT 3 potentiostat (Ametek, Berwyn, PA, USA). The measurements were performed in a standard three-electrode cell in the 0.1 M HClO_4_ solution at a temperature of 25 °C. A saturated silver chloride electrode was used as the reference electrode. A platinum wire was used as the counter electrode. The potential values were given in this study relative to the reversible hydrogen electrode (RHE).

The electrochemical characteristics of the catalysts, i.e., the ECSA and the ORR activity, were studied using the RDE (Pine Research MSR Rotator, Durham, NC, USA). For this purpose, a thin catalyst film was prepared from the catalytic “ink” based on deionized water, isopropanol, and Nafion suspension. The platinum loading at the electrode was 20 μg(Pt)/cm^2^.

Before the measurements, the catalyst was activated by cycling in the potential range of 0.04–1.0 V for 100 cycles, with a potential sweep rate of 200 mV/s at the stationary electrode in an argon atmosphere. To determine the ECSA, we recorded cyclic voltammograms (CVs) at a rate of 20 mV/s, and the charge amount consumed for the adsorption/desorption of atomic hydrogen was measured.

The ORR activity was assessed by recording potentiodynamic curves in an oxygen atmosphere at the RDE rotation speeds of 400, 900, 1600, and 2500 rpm. Further, the reaction kinetic current was calculated according to the Koutetsky–Levich equation. By dividing the resulting value by the mass of platinum deposited on the electrode and the ECSA, the values of mass and specific currents were obtained. The accuracy of determining the ECSA, as well as the specific and mass activity, was ±10%.

The catalysts’ stability was evaluated by two methods. The first method is described in [60]. For this purpose, we performed 10,000 square-wave potential cycles at 0.4 V (3 s)—1.0 V (3 s) in an O_2_ atmosphere. The catalysts’ ECSA and mass activity values were compared before and after stress testing.

The second method consisted in applying 20,000 voltametric potential sweep cycles in the range of 0.6–1.0 V [60,61]. Cycling was carried out in an argon atmosphere.

For details on the electrochemical measurements, see Supplementary Materials, Sections S3 and S4.

### 2.4. Testing of the Catalysts in Membrane Electrode Assemblies

See Section S5 in Supplementary Materials.

## 3. Results and Discussion

The analysis of the composition of the studied catalysts confirmed the information on the mass fraction of metals in the samples provided by the manufacturers (Table 1). The determination of the ratio of platinum and palladium in the PP1 and PP2 catalysts carried out by ICP AS and TXRF yielded similar results (Table 1), i.e., the composition of the metal component in both samples actually corresponds to the formula Pt_3_Pd.

**Figure 1:**
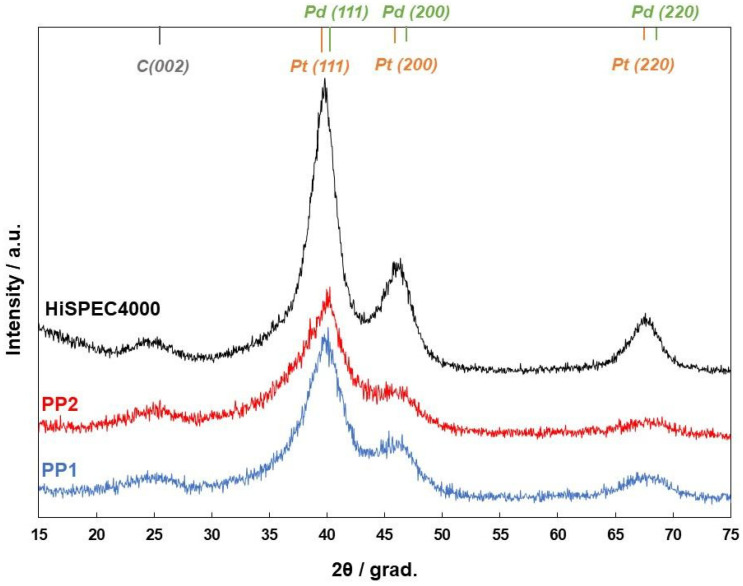
X-ray diffraction patterns of the studied catalysts (PP1, PP2, and HiSpec4000). Multi-colored segments indicate the localization of standard reflections for the facets of platinum (yellow color), palladium (green color), and carbon (black color).

We may observe characteristic reflections of the metal phase in the X-ray diffraction patterns of the catalysts (Figure 1). Platinum and palladium have a face-centered cubic (FCC) structure and exhibit similar values of the crystal lattice parameter; therefore, the positions of reflections of identical facets for these metals in the X-ray patterns are close to each other [53,54].

This makes it difficult to unambiguously interpret the diffraction patterns of bimetallic samples. At the same time, the reflections of the metal phase in the obtained diffraction patterns are localized between the positions of the reflections of platinum and palladium, which is typical for NPs with both a “solid-solution” and “core–shell” structure [62,63,64]. The X-ray diffraction patterns also show reflections in the region of 2θ = 25° corresponding to the carbon phase, which is due to the presence of a carbon support in the materials under study. It should be noted that two-phase bimetallic catalysts containing a mixture of palladium and platinum NPs can also produce similar X-ray patterns due to the superposition of close reflections of two different phases. In this regard, the X-ray data cannot be considered as unambiguous evidence of the formation of two-component NPs in the synthesis of materials.

The crystallite average size of the metal phase in the obtained bimetallic catalysts calculated by the Scherrer equation is almost the same and is equal to 2.3–2.4 nm, which is noticeably smaller than the average size of platinum crystallites in the reference sample (3.8 nm) (Table 1).

**Figure 2:**
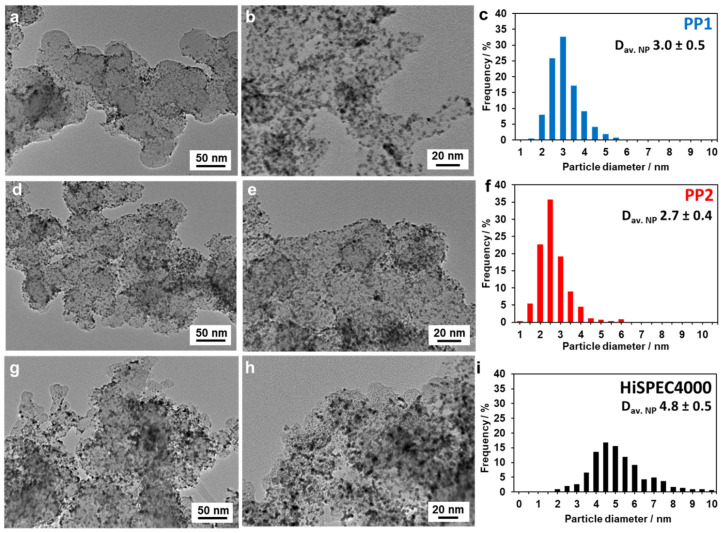
TEM micrographs of the catalysts local sections: (**a**,**b**) PP1; (**d**,**e**) PP2; (**g**,**h**) HiSPEC4000, and histograms of the NPs size distribution in the studied samples: (**c**) PP1; (**f**) PP2; and (**i**) HiSPEC4000.

The results of studying the catalysts microstructure by TEM, shown in Figure 2, correlate well with the data of X-ray powder diffraction, i.e., the average size of NPs grows in the order PP2 < PP1 << HiSpec4000. At the same time, the bimetallic PtPd/C materials demonstrate a narrower size dispersion and a more ordered spatial distribution of NPs compared to Pt/C (Figure 2).

The above results show that both bimetallic catalysts have almost the same composition of Pt_3_Pd/C and contain NPs that are smaller in size than the reference Pt/C sample. Their microstructure is also more ordered, since the range of NPs size dispersion is narrower, and their spatial distribution over the surface of the support is more uniform than those of HiSpec4000.

**Figure 3:**
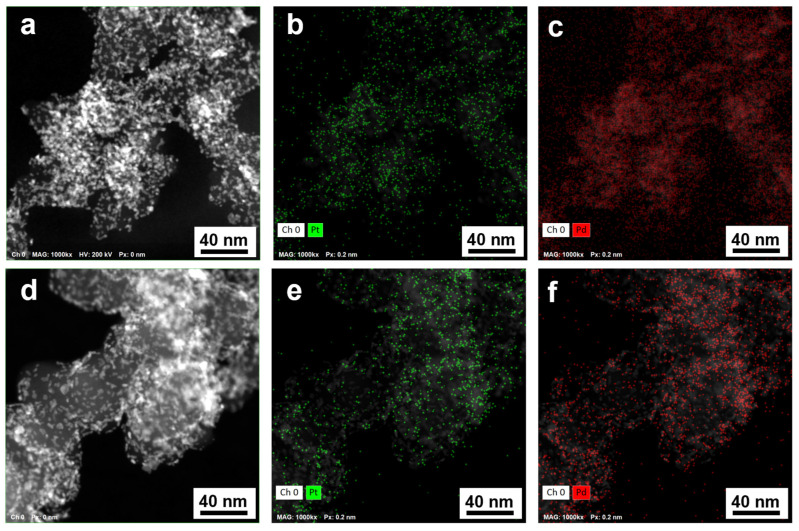
STEM image and EDX mapping of the corresponding local surface sections of the PP1 (**a**–**c**) and PP2 (**d**–**f**) catalysts: Pt—green, Pd—red.

**Figure 4:**
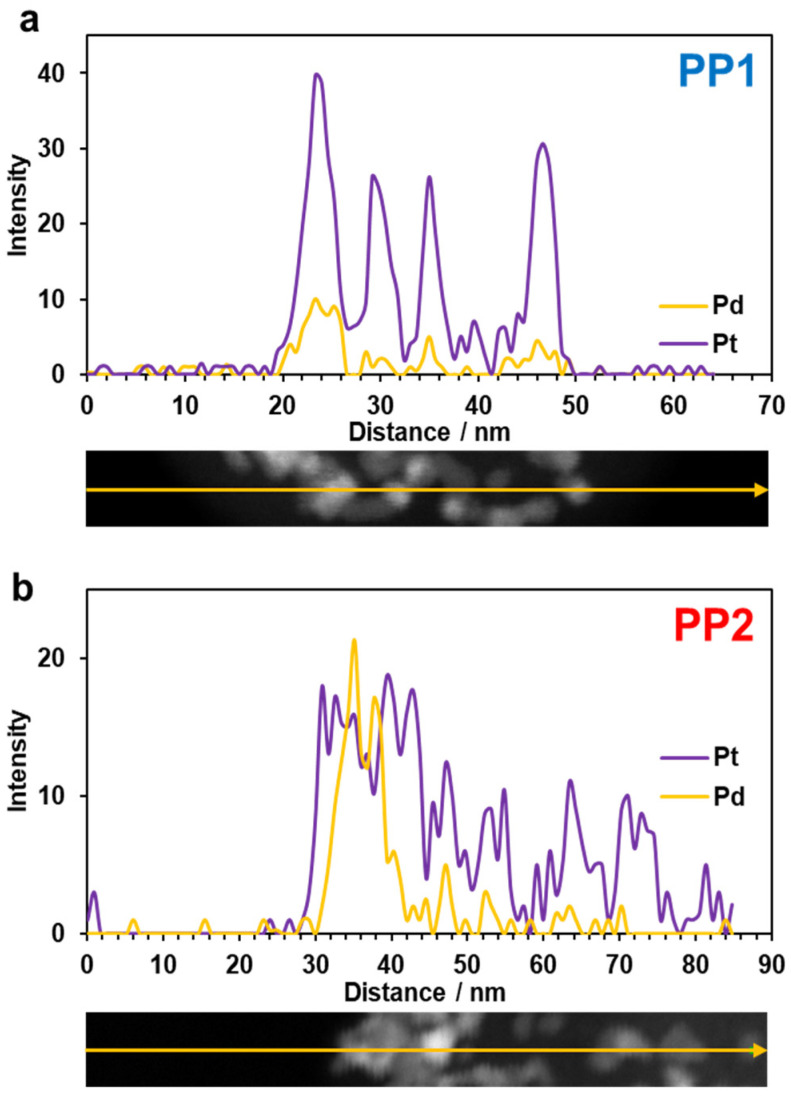
EDX analysis results: distribution of palladium (yellow) and platinum (purple) along scanning lines of the surface sections for the (**a**) PP1 and (**b**) PP2 catalysts containing several NPs. Scanning directions are indicated by yellow arrows.

The difference in the synthesis techniques for the bimetallic materials declared by the manufacturer prompted us to study in more detail the features of the distribution of metals over the surface of the support, as well as the fine structure of individual NPs. For this purpose, the EDX method was used to perform elemental mapping of the surface sections of the catalysts (Figure 3 and Figure S1), including the in-line scanning of the composition of individual NPs (Figure 4).

The results of elemental mapping for the catalysts’ surface and in-line scanning of the NP composition indicate the localization of palladium and platinum atoms in the same surface sections (Figure 3). Almost all the NPs studied are bimetallic (Figure 4a,b). In the PP2 material, we may observe particles with an increased palladium content in the core, i.e., during the in-line composition scanning of these particles, the platinum concentration in the center of NPs decreases, with the palladium concentration growing (Figure 4b). Indeed, the presence of a Pd-core—Pt-shell structure in bimetallic NPs in the PP2 catalyst appears to be most probable. During the synthesis of PP2, we initially obtained palladium NPs, some of which could be well preserved as the cores of bimetallic NPs formed upon subsequent deposition of platinum.

**Figure 5:**
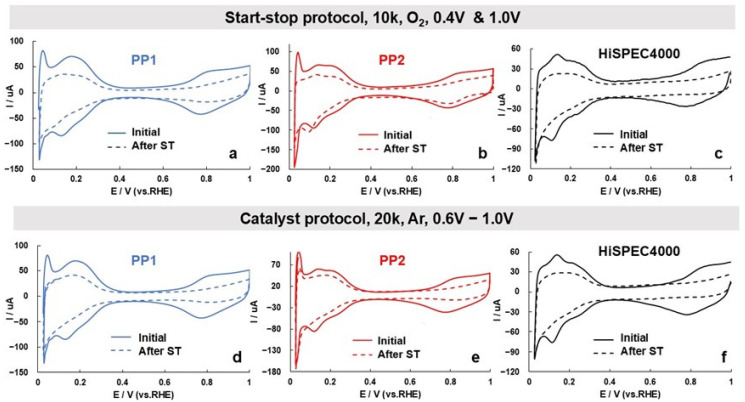
Cyclic voltammograms of the PP1 (**a**,**d**), PP2 (**b**,**e**), and HiSpec4000 (**c**,**f**) electrocatalysts before (solid line) and after (dotted line) various types of stress testing: 20,000 cycles in an Ar-saturated electrolyte at atmospheric pressure (**a**–**c**) and 10,000 cycles in an electrolyte saturated with O_2_ at atmospheric pressure (**d**–**f**). The potential sweep rate is 20 mV/s, 2nd cycle. The electrolyte is 0.1 M HClO_4_.

Taking into account the microstructure features established, one would have expected that the ECSA of the bimetallic catalysts would exceed that of the reference Pt/C sample. The ECSA value was calculated from the hydrogen region of cyclic voltammograms (Figure 5), as described in Section S4.1 of Supplementary Materials. In comparison with HiSpec4000, the CVs of both bimetallic catalysts are characterized by significantly higher currents in the oxygen and, especially, hydrogen regions, in combination with a close value of currents in the double-layer region. This indicates noticeably higher ECSA values of the bimetallic catalysts. Indeed, the calculation performed shows that the ECSA of PP1 and PP2 is 85 and 90 m^2^/g (PtPd), this value amounting to about 60 m^2^/g (Pt) for HiSpec4000 (Table 1).

**Figure 6:**
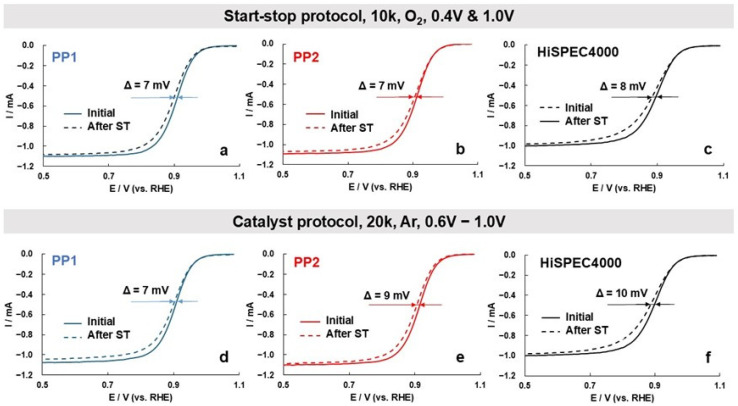
(**a**–**f**) Oxygen electroreduction linear sweep voltammograms of the PP1 (**a**,**d**), PP2 (**b**,**e**), and HiSpec4000 (**c**,**f**) catalysts before (solid line) and after (dotted line) accelerated stress testing in the following modes: 20,000 cycles in an Ar-saturated electrolyte at atmospheric pressure (**a**–**c**) and 10,000 cycles in an electrolyte saturated with O_2_ at atmospheric pressure (**d**–**f**). The potential sweep rate is 20 mV/s, 2nd cycle. The electrolyte is 0.1 M HClO_4_ saturated with O_2_ at atmospheric pressure. The disk rotation speed is 1600 rpm. The potential sweep rate is 20 mV/s.

To determine the ORR activity of the catalysts, we conducted a comparative study of the oxygen electroreduction linear sweep voltammograms (LSVs) at the RDE at rotation speeds from 400 to 2500 rpm (Figure S2a). The ORR LSVs of the bimetallic catalysts are shifted to higher potential values compared to the LSV obtained for the Pt/C catalyst, with the potentials of the oxygen half-wave growing in the order HiSpec4000 < PP1 < PP2 (Figure 6, Table 2). The straight-line sections of the dependences of *I*^−1^ on *ω*^−0.5^ for all the catalysts have the same slope corresponding to the four-electron mechanism of the ORR (Figure S2b). At the same time, the ORR mass activity of the catalysts determined by extrapolation of the straight-line segments in the Koutetsky–Levich coordinates to the *y*-axis grows in the order HiSpec4000 < PP1 < PP2 (Figure S2b). The increase in the mass activity of the bimetallic catalysts is due not only to their higher ECSA but also to the specific activity that grows in the same order (Table 2).

**Figure 7:**
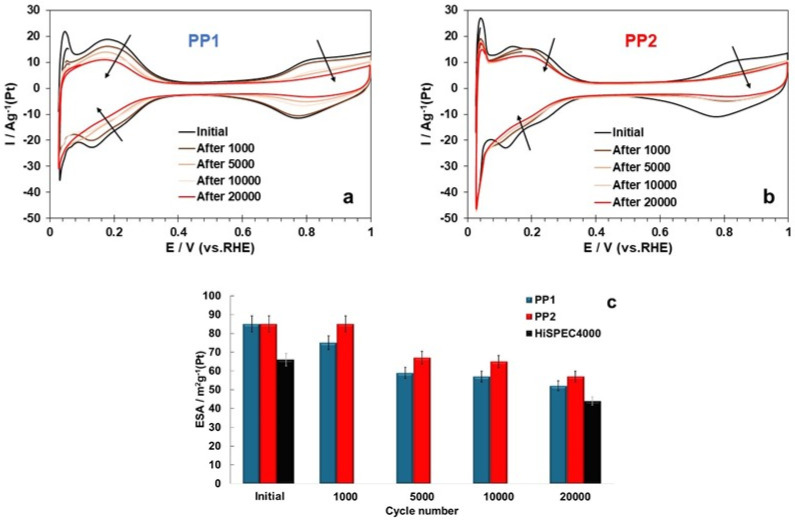
Cyclic voltammograms of the bimetallic catalysts: initial; after 1000; 5000; 10,000; and 20,000 cycles (**a**,**b**), and changes in the catalysts’ ECSA during stress testing (**c**). The cycling potential range is 0.6–1.0 V, 0.1 M HClO_4_ saturated with Ar.

Taking into account the results of a number of studies indicating a lower stability of palladium compared to platinum (see Section 1), we considered it important to compare the durability of the studied catalysts under accelerated stress testing (AST) conditions in an electrochemical cell. The use of two different AST modes showed that the stability of the catalysts differs much less than their activity in the ORR (Table 2). It should be noted that the degradation of the ECSA and the decrease in the mass activity of the catalysts after stress testing are generally not the same, especially for bimetallic catalysts. In the course of stress testing, both the size of the NPs and their shape appear to change. The former, along with the detachment and aggregation of NPs, causes a drop in the catalysts’ ECSA, while the latter affects the value of specific activity. Nevertheless, according to the results of two testing protocols, the catalysts can be placed in the following order of increasing durability: HiSPEC4000 ≤ PP1 ≤ PP2. Given the smaller NPs size and the higher ECSA values of the bimetallic catalysts, this result is rather unexpected. However, a narrower size dispersion of NPs, coupled with their more uniform spatial distribution, must make it difficult for the bimetallic catalysts to degrade through the mechanisms of Ostwald ripening and NPs aggregation [65,66,67,68]. It is not unlikely that the adhesion of platinum–palladium NPs to the support is stronger than that of platinum NPs, which also has a beneficial effect on the stability of the catalyst. Finally, the activating effect of platinum and PtPd on carbon oxidation along the perimeter of fixed NPs may be different. For example, the alloying of platinum with some d-metals is known to inhibit the high-temperature oxidation of deposited metal–carbon catalysts [69,70]. It is noteworthy that, judging by the nature of the change in the CVs and the ECSA value during stress testing, the degradation of both platinum–palladium catalysts proceeds uniformly (Figure 7).

Previously, we have already pointed out that the positive effect of the alloying with platinum on the ORR activity is usually more pronounced when comparing catalysts in solutions of sulfuric or perchloric acids in an electrochemical cell than in a membrane electrode assembly. Therefore, it was important to compare characteristics of the MEAs containing the obtained bimetallic catalysts and the HiSpec4000 commercial catalyst.

It is worth noting that an essential part of this study was compliance with equivalent testing conditions for the studied and commercial samples, i.e., the mass of precious metals deposited on 1 cm^2^ of the membrane, as well as the testing conditions of the MEAs, were the same (see Section S5). At the same time, the anode catalytic layer of all the MEAs was formed from the HiSpec4000 catalyst.

The polarization curves were measured, and the dependence of the MEA specific power on the current strength was calculated 3 (HiSpec4000) to 5 (PP2) times for each catalyst. The average values of specific power increased in the order PP1 (1221 W/g(PGM)) ≤ HiSpec4000 (1292 W/g(Pt)) < PP2 (1449 W/g(PGM)). It should be noted that when determining the ORR activity of the catalysts based on the results of measurements at the RDE in an electrochemical cell (Table 1), although the PP2 catalyst turned out to be the best, both bimetallic catalysts exhibited higher activity compared to HiSpec4000. On average, the PP1-based MEAs proved to be worse than when using HiSpec4000, although the PP2 catalyst, as in the electrochemical cell, demonstrated the highest activity.

**Figure 8:**
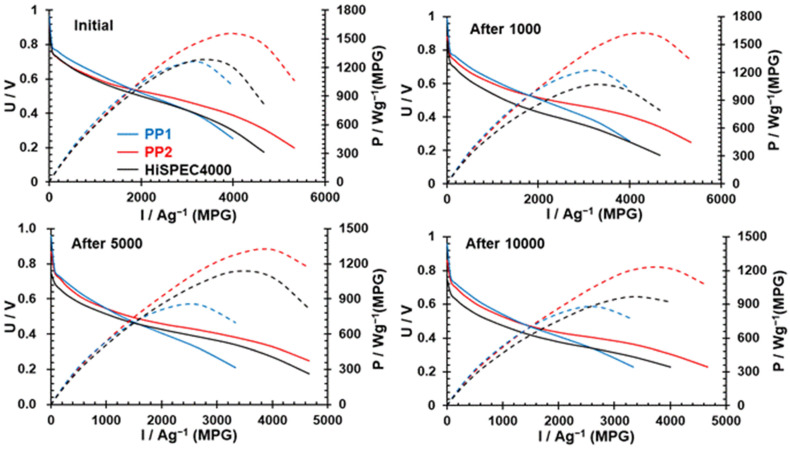
Polarization and load curves of the MEAs with HiSpec4000, PP1, and PP2 deposited on the cathode.

**Figure 9:**
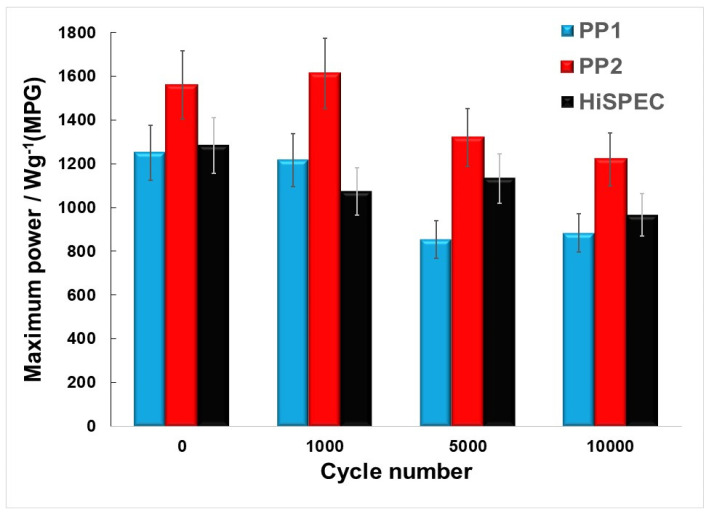
Dependence of the specific power of the MEAs with different catalysts at the cathode on the number of cycles.

The durability tests of the MEAs consisted in applying 10,000 rectangular cycles of the voltage change from 0.6 V (3 s) to 0.95 V (3 s). At the same time, we recorded polarization curves in the current range from 0 to about 4700 A/g(PGM) after 1000; 5000; and 10,000 cycles. Figure 8 shows the polarization curves, as well as the curves showing changes in the specific power of the MEAs as the current density increases, at different stages of durability testing for specific MEAs. The change in the values of maximum power density during cycling is shown in Figure 9. It can be seen from the data presented that the characteristics of the MEAs based on the PP2 cathode catalyst are not only superior at the beginning of the testing but also remain so throughout the testing in almost the entire current range. Only after long-term cycling, in the high-current region, may we observe a decrease in the differences between the catalysts (Figure 8). Under these conditions, flooding in the cathode may have the main effect on the behavior of the MEA [26].

Therefore, the MEA with a cathode based on the PP2 catalyst demonstrates higher power during durability tests compared with the MEAs based on PP1 and HiSpec4000. Before the durability tests, it exceeds the average power of the MEA_HiSpec4000_ by about 22%, this value being about 26% at the end of the testing. This means that in terms of the stability of characteristics, this MEA is not inferior to its analog using the commercial platinum–carbon catalyst (HiSpec4000) at the cathode. It is worth noting that the maximum initial specific power of the PP2-based MEA was 1560 W/g(PGM), while the PP1- and HiSpec4000-based MEAs exhibited 1333 and 1300 W/g(PGM), respectively.

## 4. Conclusions

A comparative study of the commercial bimetallic Pt_3_Pd/C catalysts PP1 and PP2 (Prometheus R&D, Russia) and the Pt/C catalyst HiSpec4000 (Johnson Matthey) containing about 40% wt. of precious metals showed that the platinum–palladium catalysts are characterized by smaller NPs with a narrower size and more uniform spatial distribution compared to Pt/C. As a result, the ECSA of the bimetallic catalysts (~85 ± 8 m^2^g^−1^ (PGM)) significantly exceeds that of Pt/C (~60 ± 6 m^2^g^−1^ (PGM)). The use of techniques for the simultaneous (PP1) or sequential (PP2) reduction of palladium and platinum precursors in the liquid phase had no effect on the composition of the catalysts. In both materials, palladium and platinum are predominantly localized at the same sections of the carbon support surface. Coupled with the results of X-ray and in-line scanning of the NPs’ composition, this confirms the formation of two-component NPs. At the same time, in the PP1 catalyst, the NPs structure predominantly corresponds to a platinum-based solid solution. In the PP2 sample, we may also observe NPs with a Pd-core—Pt-shell structure. The presence of the latter is due to the technique of sequential synthesis, i.e., being initially formed, some of the palladium NPs are preserved in the form of cores of two-component core–shell particles after the subsequent reduction of the platinum precursor.

The oxygen electroreduction reaction on all the electrocatalysts studied proceeds through a four-electron mechanism. The ORR mass activity of both platinum–palladium catalysts measured at the RDE in a three-electrode electrochemical cell turned out to be higher than that of Pt/C, increasing in the order HiSpec4000 ≤ PP1 < PP2. The higher mass activity of the bimetallic catalysts is due not only to their higher ECSA but also to their higher specific activity, which grows in the same order. Table S1 provides data on the activity of the platinum–palladium catalysts in the ORR derived from various literature sources. These catalysts are obtained on the basis of different carbon supports or represent metal materials that contain no carbon. Unfortunately, we do not have the opportunity to analyze the methods of preparation of the catalytic layers, nor the features of the methods to study the catalysts’ electrochemical behavior and process the results of such measurements. Obviously, they differ significantly in various works, and this should be taken into account when comparing the given values of mass activity. Nevertheless, only one of the catalysts shown in the table demonstrated a higher ORR mass activity than the commercial PP2 catalyst studied in this work.

The rate of degradation of the catalysts determined by accelerated stress testing methods in a three-electrode electrochemical cell increased slightly in the order PP2 < PP1 ≤ HiSpec4000, although the durability of the catalysts did not differ as significantly as their activity in the ORR.

Testing of the catalysts at the cathode of MEAs for hydrogen–air fuel cells demonstrated the similarity of the characteristics of Pt/C and PP1 and confirmed the higher efficiency of the PP2 Pt_3_Pd/C catalyst. The positive features in the behavior of this catalyst in the electrochemical cell and in the membrane electrode assembly correlate well with one another.

The results of this study are believed to indicate the prospects of using bimetallic PtPd/C materials as catalysts in PEMFCs. Considering that the Pt_3_Pd/C catalysts were not subjected to the optimization of the catalytic ink composition and the conditions for the formation of the catalytic layer when applying the latter to the membrane, the prospect of increasing the power characteristics of the PEMFC MEAs by 20% in the case of their use seems quite possible. The platinum–palladium catalysts obtained by sequential deposition of palladium and platinum and containing bimetallic NPs with a Pd-core—Pt-shell structure appear to be more promising. An attempt to obtain catalysts based on the NPs with a core–shell structure (but with a higher palladium content than 1:3) seems to be a quite interesting trend for continuing research.

## Figures and Tables

**Table 1:** Values of the parameters characterizing the composition, microstructure, and electrochemical behavior of the studied catalysts. Values averaged on the basis of several measurements.

Sample	Metals Loading, % wt.	Composition		D_Av_ (cryst.), nm	D_Av_ (NPs), nm	ECSA, m^2^/g (PGM)	MA, A/g (Pt)	SA, A/m^2^	E_1/2_ at 1600 rpm, V
		TXRF	ICP AS						
PP1	37.5	Pt_78_Pd_22_	Pt_73_Pd_27_	2.3	3.1	85	322	3.8	0.90
PP2	38.2	Pt_75_Pd_25_	Pt_74_Pd_26_	2.4	2.6	90	407	5.0	0.91
HiSpec4000	40.0	Pt	Pt	3.8	4.8	60	243	4.0	0.90

**Table 2:** Values of the parameters characterizing the electrochemical performance of the catalysts.

Sample	Before ST				Arter ST				Degree of Degradation (ECSA), %	Degree of Degradation (MA), %
	ECSA, m^2^/g (PGM)	Surface Activity, A/m^2^	Mass Activity, A/g (PGM)	E1/2, V	ECSA, m^2^/g (PGM)	Surface Activity, A/m^2^	Mass Activity, A/g (PGM)	E1/2, V		
Start–stop protocol (O_2_, 10 k cycles, 0.4 & 1.0 V)										
PP1	87	3.5	304	0.90	50	4.2	212	0.90	43	30
PP2	91	4.4	403	0.91	59	5.0	295	0.90	35	27
HiSPEC	66	3.4	226	0.90	34	4.0	135	0.89	47	43
Catalyst protocol (Ar, 20 k cycles, 0.6–1.0 V)										
PP1	85	3.6	304	0.90	52	4.4	228	0.90	39	25
PP2	85	4.9	420	0.90	57	5.4	307	0.90	33	27
HiSPEC	66	3.2	212	0.90	44	3.0	133	0.88	33	37

## Data Availability

Data is contained in the article and Supplementary Materials. Any additional data and explanations to them are available on request from the corresponding author.

## Supplementary Materials

The following supporting information can be downloaded at: https://www.mdpi.com/article/10.3390/nano14201672/s1, Table S1. Comparison of the ECSA and mass activity values for the present Pt_3_Pd/C electrocatalysts and some other PtPd-based materials [49,50,51,53,54,71,72,73]; Figure S1. STEM image and EDX mapping of the corresponding local surface sections of the PP1 (a–c) and PP2 (d–f) catalysts; Figure S2. (a) Oxygen electroreduction polarization curves measured for PP2 at different rotation speeds of the disk electrode and (b) Koutetsky–Levich dependences at a potential of 0.90 V for PP1, PP2, and HiSpec4000; Table S2. Results of determining the power characteristics of the MEA based on PP2 during 30,000 testing cycles; Materials and Methods (Supplement); Figure S3. Photograph of the fixture with a clamped and dried membrane (a) and the fixture with catalytic layers deposited onto the membrane (b); Table S3. Step-load measurement intervals for the MEA; Table S4. Sequence of actions to recover reversible losses of the MEA.
