# Supplementary material for: Exploring the Potential of Bimetallic PtPd/C Cathode Catalysts to Enhance the Performance of PEM Fuel Cells
Source: Nanomaterials (Basel). 2024 Oct 18;14(20):1672. doi: 10.3390/nano14201672 (PMC11510532; doi:10.3390/nano14201672)
Supplement: Supplementary file 1 [file nanomaterials-14-01672-s001.zip › nanomaterials-3258129-supplementary.pdf]

Supplementary Materials

Exploring the Potential of Bimetallic PtPd/C Cathode Catalysts to Enhance the Performance of PEM Fuel Cells

Vladimir Guterman [1,*], Anastasia Alekseenko [1], Sergey Belenov [1,2], Vladislav Menshikov [1,2], Elizaveta Moguchikh [1], Irina Novomlinskaya [1,2], Kirill Paperzh [1] and Ilya Pankov [3]

1 Faculty of Chemistry, Southern Federal University, 7 Zorge St., Rostov-on-Don 344090, Russia; aalekseenko@sfedu.ru (A.A.); sbelenov@sfedu.ru (S.B.); vmenshikov@sfedu.ru (V.M.); moguchih@sfedu.ru (E.M.); igerasimova@sfedu.ru (I.N.); paperzh@sfedu.ru (K.P.)
2 Prometheus R&D LLC, 4g/36 Zhmaylova St., Rostov-on-Don 344091, Russia
3 Research Institute of Physical Organic Chemistry, Southern Federal University, 194/2 Stachki St., Rostov-on-Don 344090, Russia; ipankov@sfedu.ru
* Correspondence: guter@sfedu.ru; Tel.: +7-9045001050

Table S1. Comparison of the ECSA and mass activity values for the present Pt3Pd/C electrocatalysts and some other PtPd-based materials (NA—data is not available).

Electrocatalyst	PtPd loading, % wt.	ECSA, $m^2/g(PtPd)$	Mass activity, mA/mg	Reference link
**PP2**	**38.2**	**90**	**407**	**This work**
PP1	37.5	85	322	**This work**
Pt_3Pd_1 on ordered mesoporous carbon	10	NA	354	https://doi.org/10.1016/j.apsusc.2018.12.072
Pt_1Pd_1 on ordered mesoporous carbon	10	NA	286	https://doi.org/10.1016/j.apsusc.2018.12.072
PtPd on graphitic carbon nanofibers	NA	44.6	420	https://doi.org/10.1016/j.apcatb.2019.118080
$Pt_{14}Pd_{86}/C$	30	49	NA	https://doi.org/10.1016/j.jpowsour.2009.09.006
PtPd/rGO	19.4	NA	139	https://doi.org/10.1016/j.electacta.2015.12.068
NPs-$Pt_{75}Pd_{25}$	40	NA	250	https://doi.org/10.1016/j.electacta.2014.11.160
PtPd nanoflowers	100	12.2	120	https://doi.org/10.1016/j.electacta.2014.06.052
Pd_3Pt_1/XC-72R	20	26	8.64 at 0.85 V	https://doi.org/10.1016/j.apcatb.2010.04.015
Pd_3Pt_1 on ordered mesoporous carbon	20	29	14.02 at 0.85 V	https://doi.org/10.1016/j.apcatb.2010.04.015
Pd@Pt core–shell tetrapods	100	20.1	381	https://doi.org/10.1039/C4TA04917A

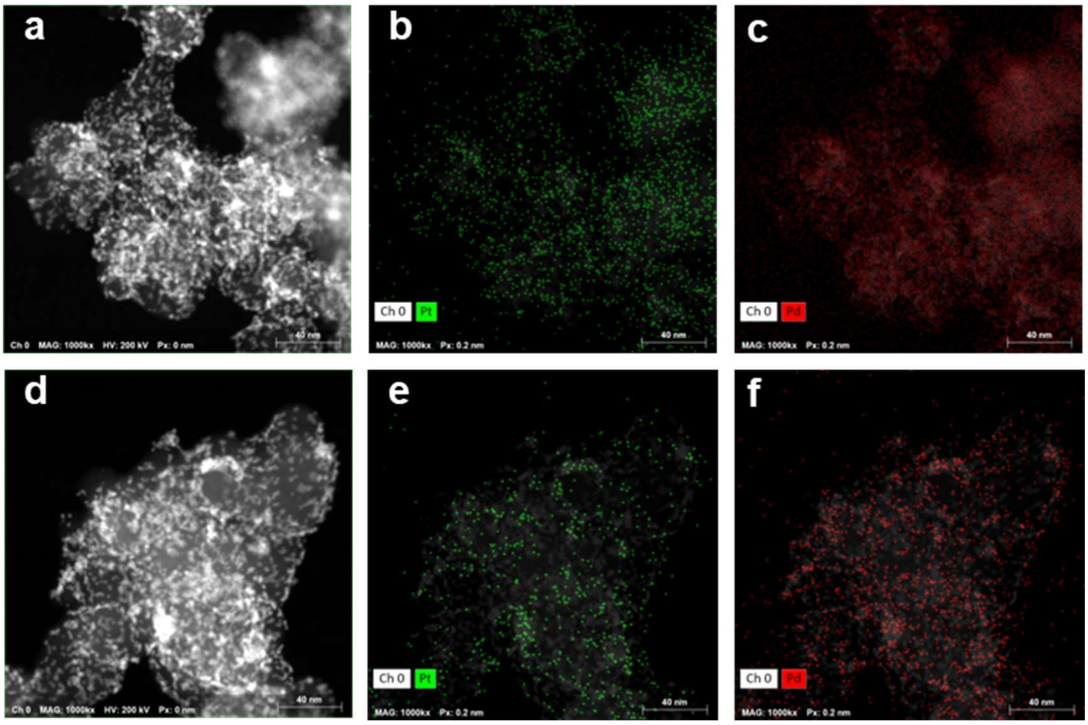

Figure S1. STEM image and EDX mapping of the corresponding local surface sections of the PP1 (a–c) and PP2 (d–f) catalysts. Pt—green, Pd—red.

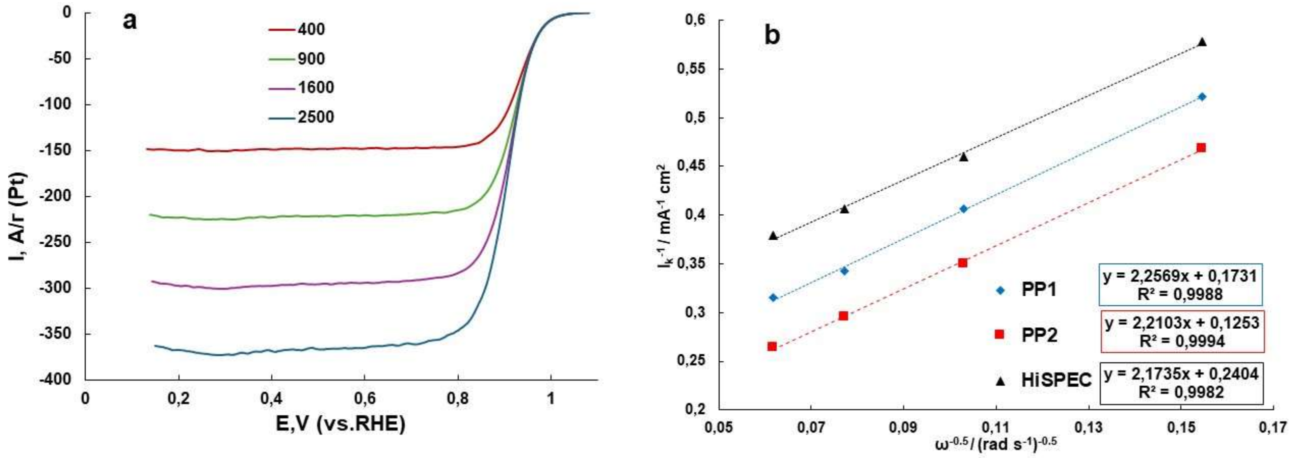

Figure S2. (a) Oxygen electroreduction polarization curves measured for PP2 at different rotation speeds of the disk electrode and (b) Koutetsky–Levich dependences at a potential of 0.90 V for PP1, PP2, and HiSpec4000.

For the MEA based on the PP2 catalyst, which exhibited the highest characteristics, we performed one stress test with 30,000 voltage overlay rectangular cycles at 0.6 and 0.95 V in an argon atmosphere. The test results presented in Table S2 confirm the previously drawn conclusions about a gradual decrease in the power of the MEA and the relatively high durability of the catalyst and catalytic layers.

Table S2. Results of determining the power characteristics of the MEA based on PP2 during 30,000 testing cycles.

Material	P_{max}, W/g(PGM)					Degradation in power, %
	Initial state	1,000 cycles	5,000 cycles	10,000 cycles	30,000 cycles	
**PP2**	1,480	1,470	1,230	1,027	840	43

Materials and Methods (Supplement)

S1. Reagents used

The following chemicals and materials were used in the experimental work: distilled H_2O (conductivity <5 μS/cm, GOST 58144–2018), perchloric acid $HClO_4$ (extra pure, Sigma-Aldrich), 10% aqueous Nafion® solution D1020, isopropyl alcohol (extra-pure grade), argon (Ar, 99.9%, Globus, Moscow, Russia), O_2 (from electrolyzer).

S2. Study of the materials composition and microstructure

S2.1. Gravimetric analysis

The gravimetry method was used to determine the mass fraction of metals contained in the catalysts. Ceramic crucibles were precalcined in a muffle furnace (UED-7-10D, Russia) at a temperature of 800 ℃. After complete cooling, the crucibles were weighed by fixing the mass ($m_{crucible}$) and filled with a weighed amount of the catalyst ($m_{catalyst} \approx 0.02$ g). Further, the crucibles were again immersed in the furnace at the same temperature. After 40 min, the crucibles with the metal component were removed from the furnace and cooled to room temperature in a desiccator over P_2O_5, after which the mass of the crucible with the unburned residue ($m_{crucible+res}$) was recorded. The mass fraction of metals was calculated using the following formula:

$$\omega = (m_{crucible+res} - m_{crucible})/m_{catalyst} \times 100\% \quad (S1)$$

S2.2. Study of the catalysts structural characteristics by X-ray powder diffraction

The ARL X`TRA diffractometer (Thermo Scientific, USA) with a Bragg–Brentano geometry (θ-θ) and a CuKα radiation (λ = 0.154056 nm) at room temperature was used to record X–ray patterns. X-ray patterns of the studied samples were recorded in the angle range of $15° \leq 2\theta \leq 55°$ by step-by-step scanning with a detector increment of 0.02. The X-ray patterns were processed using the SciDAVis software.

S2.3. Determination of the chemical composition by total reflection X-ray fluorescence

A Pt:Cu ratio in the samples was determined by TXRF using the RFS-001 spectrometer with a total external reflection of the X-ray radiation (Research Institute of Physics, Southern Federal University, Rostov-on-Don). The spectrum acquisition time was 300 s. The X-ray fluorescence spectra were recorded and processed using the UniveRS software (Southern Federal University, Rostov-on-Don).

S2.4. Determination of the chemical composition by inductively coupled plasma atomic emission spectroscopy

The mineralization of the samples was carried out on an electric stove at a temperature of 200 °C for 2 h with a mixture of concentrated hydrochloric and nitric acids (aqua regia) in the Milestone Ethos-1 microwave mineralization unit. The measurement was performed using the ICP AC spectrometer Thermo iCap 7400Duo. The calibration of the ICP AC spectrometer was performed using a multi-element standard containing the components to be determined.

S2.5. Study of the material microstructure based on the results of transmission electron microscopy

The samples microstructure features were studied by TEM using the JEOL JEM F200 microscope (JEOL, Japan) with an attachment for elemental mapping of the sample surface section. The sample preparation was performed by preparing a catalyst suspension in isopropanol and then applying an aliquot of 3 μL to the copper grid intended for measurements. Histograms of the NPs size distribution in the catalysts were plotted based on the results of determining the size of at least 200 particles randomly selected in the TEM micrographs at various sections of the sample using Digimizer Image Analysis Software.

S3. Catalytic ink preparation technique and formation of the catalytic layer at the end face of the rotating disk electrode

The catalyst suspension (catalytic ink) was obtained by adding 3,600 μL of isopropyl alcohol, 300 μL of deionized water, and 100 μL of 1% Nafion® D1020 to 0.0060 g of the catalyst. The suspension was then dispersed in an ultrasonic bath with a frequency of 35 kHz (power 100 V) for 25 min, after which it was stirred for 5 min with a mechanical magnetic stirrer. The total dispersion time required to achieve homogeneity of the catalytic ink was at least 30 min.

Before applying the suspension, the glass–carbon end face of the RDE was polished in accordance with the method recommended by the manufacturer (see Pine Research Electrode Polishing Guide https://pineresearch.com/shop/products/electrodes/rde/classic-ptfe/e3/#documentation and https://www.youtube.com/watch?v=B1vndNRUnV4&t=39s) and then rinsed in isopropyl alcohol.

An aliquot of the ink with a volume of 3.1 ± 0.1 μL was sampled with a pipette tip with continuous stirring on a magnetic stirrer. A drop of the ink was applied to the end face of the glass–carbon electrode with a diameter of 5 mm (area 0.196 cm^2) mounted vertically on the scales. The ink mass was monitored. Next, the electrode was dried with rotation in an upright position at a speed of 700 rpm until the drop was completely dry (10–15 min).

After drying the first drop of the catalytic ink, a second drop of the same volume was applied in the manner described above. In these conditions, the total volume of the applied ink was 6.2± 0.2 μL, with the metal loading at the RDE end face being in the range from 18.0 μg/cm^2 to 21.0 μg/cm^2.

The electrode thus obtained was a homogeneous porous catalyst layer fixed to the glass–carbon end face of the disk electrode.

After being used, the catalytic ink was stored at a temperature of 10–25 °C in a glass container with a tightly closed lid.

S4. Electrochemical study methods (voltammetry)

The electrochemical measurements were performed in a three-electrode cell using the VersaSTAT potentiostat (AMETEK Scientific Instruments, USA) and the rotating disk electrode (Pine Research Instruments, USA). A saturated silver chloride electrode connected to the electrochemical cell by means of a salt bridge was used as the reference electrode. A platinum wire was used as the auxiliary electrode. All potentials used in this work were adjusted to the RHE.

S4.1. Determination of the electrochemically active surface area

The electrolyte (0.1 M $HClO_4$) was saturated in the cell with argon for 30 min. After that, the surface of the working electrode was standardized by cyclic voltammetry by setting 100 potential sweep cycles in the range from 0.025 to 1.00 V (relative to the RHE), with a potential sweep rate of 200 mV/s.

To determine the ECSA, we recorded 3 cyclic voltammograms at the stationary electrode in the potential range of 0.025–1.00 V and with a potential sweep rate of 20 mV/s. The ECSA was calculated for the second voltammogram by the half-sum of the charge amounts consumed for the adsorption (Q′) and desorption (Q″) of an atomic hydrogen monolayer according to the formula:

$$ESA = \frac{(Q' + Q'')/_2}{m_{МПГ} * 0,2065}, \quad \text{(S2)}$$

where m_{PGM} is the metal loading at the electrode, and 0.2065 mC/cm^2 is the charge amount required to oxidize the atomic hydrogen monolayer per 1 cm^2 of the platinum surface.

S4.2. Determination of the catalysts' activity in the oxygen electroreduction reaction (linear sweep voltammetry to determine the activity)

After determining the ECSA (Clause 4.1), we assessed the ORR activity of the catalysts. For this purpose, the background potentiodynamic polarization curve was measured in an argon-saturated electrolyte at a rotation speed of 1,600 rpm with a potential sweep from 0.05 to 1.1 V at a rate of 20 mV/s. The electrolyte was replaced with a freshly prepared one purged with oxygen for 1 h, after which the potentiodynamic curves were re-recorded with a potential sweep from 0.05 to 1.1 V at a rate of 20 mV/s at four disk electrode rotation speeds: 400; 900; 1,600; and 2,500 rpm. The LSV curves were replotted taking into account the resistance of the solution (iR compensation) and the subtraction of the curves measured in an argon-saturated electrolyte solution.

The straight-line dependences in the Koutetsky–Levich coordinates (dependence of I^{-1} on $\omega^{-0.5}$) were plotted using current values at a potential of 0.9 V. By extrapolating each straight-line dependence to the y-axis, the kinetic current value (mass activity) was calculated. By the slope ratios of the straight lines, we determined the number of electrons involved in the electroreduction of the oxygen molecule. The value of the half-wave potential was determined for LSVs measured at a rotation speed of 1,600 rpm ($E_{1/2}$).

S4.3. Assessment of the catalysts stability by accelerated stress testing

S4.3.1. Protocol by DOE Durability Working Group dated 10/4/2011 (https://www.energy.gov/eere/fuelcells/articles/rotating-disk-electrode-aqueous-electrolyte-accelerated-stress-tests-pgm)

Step 1. The electrolyte (0.1 M $HClO_4$) in the electrochemical cell was saturated with argon for 30 min. After that, the surface of the working electrode was standardized by cyclic voltammetry by setting 100 potential sweep cycles in the range from 0.025 to 1.00 V, with a potential sweep rate of 200 mV/s.

Step 2. Three cyclic voltammograms were recorded in the potential range of 0.025–1.00 V at a potential sweep rate of 20 mV/s. The initial $ECSA_0$ was calculated from the half-sum of the charge amounts consumed for the adsorption and desorption of a monolayer of atomic hydrogen in the second CV. Similarly, the ECSA values after 1,000; 5,000; 10,000 and the resulting value of $ECSA_{20,000}$ after 20,000 stress testing cycles were determined.

Step 3. The initial ORR activity of the catalysts (I_0) was evaluated as described in Section S4.2.

Step 4. The electrolyte was replaced with a fresh portion saturated with argon for 30 min, after which repeated cycling was performed at the stationary electrode in the potential range of 0.6–1.0 V, with a sweep rate of 100 mV/s, over a total of 20,000 cycles.

Step 5. The ORR activity of the catalysts upon completion of the stress testing was evaluated in the same manner as described in S4.2.

The value of the degree of catalysts degradation (DD) during and after the stress testing was calculated from a change in the ECSA and the ORR mass activity according to the formulas:

degradation by ECSA = $((ECSA_0 - ECSA_N)/ECSA_0) * 100\%$

degradation by mass activity = $((I_0 - I_{20,000\ cycles})/I_0) * 100\%$.

S4.3.2. Start–stop stability assessment protocol

The protocol is described in the report by Toyota (https://iopscience.iop.org/article/10.1149/2.0161907jes).

Step 1 and Step 2 are the same as in Section S4.3.1.

Step 3: the ORR activity was measured in the same manner as described in Section S4.2.

Step 4. The stress testing was conducted in an oxygen-saturated electrolyte at the stationary electrode by sequentially setting potentials of 0.4 and 1.0 V with an exposure time of 3 s at each value. The number of cycles was 10,000, the duration of the test amounting to about 17 h.

Step 5: upon completion of the stress testing, the electrolyte was replaced with a fresh one, saturated with argon for 30 min, after which the ECSA measurement was performed, as described in Section S4.1.

Step 6. The resulting value of the ORR mass activity was determined as described in Section S4.2.

The degree of degradation as per the ECSA and the ORR mass activity (Figure 12) was calculated using the following formulas:

Degradation by ECSA = $((ECSA_{initial} - ECSA_{after\ AST})/ECSA_{initial}) * 100\%$

Degradation by mass activity = $((I_{initial} - I_{after\ AST})/I_{initial}) * 100\%$.

S5. Testing in a single membrane electrode assembly

S5.1. Catalytic ink preparation technique

S5.1.1. Preparation of the catalytic ink for the membrane electrode assembly cathode

For the preparation of the cathode ink and the formation of the MEA with an active area of 5 cm^2 with a PGM loading in the catalytic layer of 0.3 mg(PGM)/cm^2, it was necessary to take into account the inevitable losses of the catalyst during the formation of the catalytic layer by spraying, which amounted to 20%. Therefore, the above calculations include a 20% excess of reagents by weight and volume.

An amount of 450 μL of deionized water, 225 μL of isopropyl alcohol (extra-pure grade), and 20 μL of a 10% aqueous Nafion® solution were added to the catalyst suspension containing 1.8 mg of the PGM. Then, for 1.5 h, we performed ultrasonic homogenization of the ink in an ultrasonic bath with an operating frequency of 35 kHz at a water temperature not exceeding 25 °C. Mechanical shaking was additionally performed every 15–20 min for 1–2 min.

The Nafion-to-carbon mass ratio in the catalytic layer was 7:10 (0.7).

S5.1.2. Ink preparation technique for the commercial electrocatalyst used at the membrane electrode assembly anode

For the preparation of the anode ink and the formation of the MEA with an active area of 5 cm^2 with a PGM loading in the catalytic layer of 0.4 mg(Pt)/cm^2, it was necessary to take into account the inevitable losses of the catalyst during the formation of the catalytic layer by spraying, which amounted to 20%. The above calculations include a 20% excess of reagents by weight and volume.

An amount of 600 μL of deionized water, 300 μL of isopropyl alcohol (extra-pure grade), and 25 μL of a 10% aqueous Nafion® solution were added to the catalyst suspension weighing 0.0060 g and containing 2.4 mg of Pt.

After that, for 1.5 h, we performed ultrasonic homogenization of the ink in an ultrasonic bath with an operating frequency of 35 kHz at a water temperature not exceeding 25 °C. Mechanical shaking was additionally performed every 15–20 min for 1–2 min.

The Nafion-to-carbon mass ratio in the catalytic layer was 7:10 (0.7).

S5.2. Preparation of the membrane for spraying

The protective films were removed from both sides of the membrane cut to the size of the MEA, after which the membrane was weighed. Next, the membrane sample was soaked in deionized water saturated with argon at atmospheric pressure and stored in a dark place in a closed container for at least 24 h. After 24 h of exposure, the wet membrane was clamped in a special equipment and kept in a desiccator over the saturated $MgCl_2$ solution for at least 12 h, which was necessary to remove excess moisture from the membrane surface (Figure S3a).

S5.3. Spraying of the catalytic ink onto the membrane

The dried proton-exchange membrane, which was in a clamped position, was placed on a horizontal platform heated to 90 °C. The catalytic ink was sprayed alternately on both sides of the membrane (Figure S3b). The JAS 1186 spraying gun with a 0.2 mm nozzle diameter was used for spraying. The mass of the membrane with deposited catalyst layers was monitored by weighing.

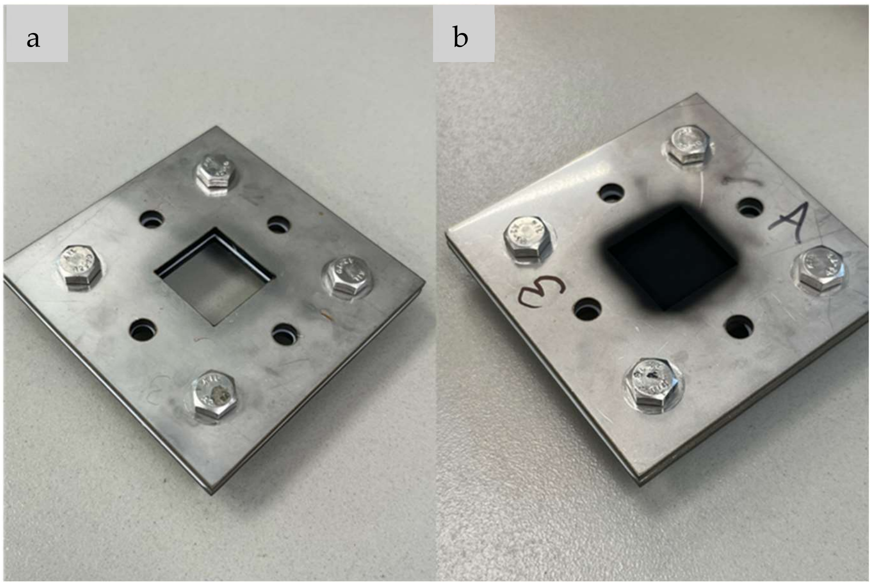

Figure S3. Photograph of the fixture with a clamped and dried membrane (a) and the fixture with catalytic layers deposited onto the membrane (b).

S5.4. Assembling of a single MEA

The MEA was assembled by hot pressing. For this purpose, the membrane with deposited catalytic layers was placed between sheets of the gas diffusion layer (Toray, TGP-H-60). Next, the resulting MEA was placed between two insulation Teflon gaskets with a thickness of 200 µm, preliminarily wiped with acetone, and transferred under the press. The assembly pressing was carried out for 3 min at a temperature of 130 °C and a pressure of 80 kg/cm^2. After pressing, the assembly was cooled at room temperature for 10–15 min between two metal plates with no pressure.

S5.5. Storage of the catalytic ink and the resulting MEA

The catalytic ink was stored in a glass container with a tightly closed lid at a temperature of 10–25 °C. The shelf life of the catalytic ink did not exceed 24 h.

The ready-to-test MEA was stored in a desiccator over the saturated $MgCl_2$ solution for no more than 72 h.

S5.6. Study of the catalytic layers activity in the membrane electrode assembly

The performance of the cathode catalyst in a single PaxiTech cell with an active area of 5 cm^2 was studied at the BioLogic FCT-50S station. The gas pressure was 1.5 bar; the temperature of the gas channels was 80 °C; and the cell temperature was 75 °C. The anode gas was hydrogen, 99.999% pure. The cathode gas was filtered air (primary filtration from dust), with an oxygen content of 21%. The flow rate of the anode gas was set at 220 mL/min, this value being set at 680 mL/min for the cathode gas.

After that, we applied a current of 0.6 A/cm^2 thereto and waited for (no more than 4 h) the moment until the fluctuations in the cell potential exceeded ±5 mV for 15 min. The step load was then set according to Table S3. The load curves were measured at least twice. In the event of a difference in the maximum power of more than 10%, the results were reproduced for the third time. In this regard, each reproduction was performed using a new MEA, with freshly applied catalytic layers.

Table S3. Step load measurement intervals for the MEA.

Interval	Current density, A/cm^2	Duration, min
1	0.2	3
2	0.4	3
3	0.6	3

4	0.8	3
5	1	3
6	1.2*	3
7	1.4*	3
8	1.6*	3
9	1.8*	3
10	2*	3
11	1.8*	3
12	1.6*	3
13	1.4*	3
14	1.2*	3
15	1	3
16	0.8	3
17	0.6	3
18	0.4	3
19	0.2	3
20	0.1	3
21	0.05	3
22	0.02	3
23	0.05	3
24	0.1	3
25	0.2	3

*—if the MEA voltage at this interval was less than 0.2 V, then this and the following intervals were skipped, and the measurements were continued in the opposite direction from the last set current density value.

S5.7. Study of the catalytic layers stability during 10,000 cycles

After measuring the activity of the catalytic materials, as indicated in Clause 5.6., the stability of the cathode catalyst was evaluated. The gas pressure was atmospheric; the temperature of humidifiers, gas lines, and the cell was 80 °C. The anode gas was hydrogen, 99.999% pure; the cathode gas was argon, 99.99% pure. The flow rate of the anode gas was 200 mL/min, this being 75 mL/min for the cathode gas. In each cycle, we set a rectangular voltage pulse at 0.6 V (3 s) and 0.95 V (3 s). The total number of cycles was 10,000. In the course of cycling, after 1,000; 5,000; and 10,000 cycles, we returned reversible losses according to Table S4 and measured load curves according to the procedure described in Clause 5.6.

Table S4. Sequence of actions for the return of reversible losses of the MEA.

Intervals	Anode gas	Anode gas flow rate, mL/min	Cathode gas	Cathode gas flow rate, mL/min	Duration, s
1	Argon	200	Argon	400	120
2	-	0	Air	400	900
3	Argon	200	Argon	400	120
4	H_2	200	-	0	600
5	H_2	200	Air	400	5

The stability of the cathode catalyst in the MEA was assessed by a change in the maximum power and voltage at set current densities after 1,000; 5,000; and 10,000 cycles.
